# Richardson’s Anatomy

**Published:** 1854-08

**Authors:** 


### RICHARDSON'S ANATOMY.

We have already given the impressions of two reviewers respecting this work, but cheerfully make room for the following notice from the pen of a promising young anatomist of our city.

We allude, with pleasure to this work of Dr. Richardson. Months ago, when we saw this anatomist looking over files of manuscript, we predicted the out-coming of an anatomy which would do credit to its author. It is written for students, for the *dissecting rooms*, the wants of which few can be presumed to know better that Dr. Richardson. It has not the minutiæ of Cruveilhier, or of Quain and Sharpey, but for reference for the surgeon it will be found all that is desired. The chapter on general anatomy supplies, we think, a desideratum. It is a general and finite view of microscopic anatomy. He describes here what is *visible*, and what he himself has seen. Not so much as usual is said of osteogeny—this is well, amply enough is given for an elementary book. With the myology, splanchnology, anginology and neurology we are alike pleased, and we like the *practical* order in which they are severally treated of. Every thing is written in a clear, concise, readable manner.

In speaking of the capacity of blood-vessels, the author alludes to and corrects the remarkable error, found in some not very old works on anatomy, relative to the increase of capacity of vessels as they divide. False physiological deductions as to the increasing velocity of the circulating fluid, &c., of more importance than the error itself, have hence arisen. We regret that Dr. Richardson did not proceed farther, and state more exactly the quantity and law of this increase in the capacity of arteries and veins. We doubt not that nature will be found here recognising, as it were, her hydraulic and hydrostatic principles. We doubt not, that when division of an arterial trunk occurs, the diameters of the branches are determined by the length of the tube, its tortuosity or directness, its direction, horizontal or perpendicular, the moderate or extraordinary *vires à térgo et a frontè* of the blood—in fine, by all the laws of hydraulics and hydrostatics. Let us take for example the aorta at its termination. We obtain the following data from measurement:

| | | Inches. | | | Sq. Inches. |
|---|---|---|---|---|---|
| Diameter of | Aorta, | .7766 | Area of | Aorta, | .4737 |
| do | Iliacs, | .5093 | do | Iliacs, | .2037 |
| do | Middle Sacral, | .1012 | do | Middle Sacral, | .0081 |

It will be observed that the sum of the areas of the terminal branches exceeds the area of the trunk by .01511 square inches. So it has been inferred that the velocity of the blood was slightly diminished. But though the area of the aorta is less than the sum of the area of its branches, its power of transmission of fluids is greater than theirs. Its power, in this respect, is to the power of each iliac 29.10; the power of the aorta is to that of the middle sacral as 731.10; and the power of the aorta is to the sum of the powers of its terminal branches as 50.35 (approximatively). It will be seen that in this calculation several of the principles, which we have stated as determining the calibre of blood vessels at their divisions, are disregarded; otherwise we believe an exact adaptation, in size, of the aorta to its terminal branches would be shown.

Dr. Richardson, with all other anatomists whose works we have seen, considers the action of the psoas-iliac muscle to be rotation outward of the thigh. We venture to express our doubts as to the accuracy of this statement. The muscle, in passing to its insertion, crosses over the head of the femur. When the muscle contracts, the power exerted indirectly from before backward, on the head, is sufficient at least to counterbalance the direct force of the muscle applied upon a shorter lever.

The description of the peritoneum is not sufficiently minute. Doubtless the author described it in the way he considered the most useful. To some it may appear that this description ought, in consideration of its want of practical interest, to be passed over in a general way. We think a demonstration of so intricate a structure, if undertaken, should be perfect. Nothing should be left to the imagination of the reader. Who, upon reading most carefully the description of the peritoneum, as it is found in most works on anatomy, is not irritated, when after patiently following the author through with his labarynthian reflections, he is told that it is a shut sac, in which the author himself has left a gaping void? Who can conceive of the cavity of the lesser omentum as a shut sac, capable of inflation, when in the description no mention is made of the reflection of peritoneum frcm the 'left extremity' of the lesser omentum to the gastrosplenic omentum, spleen and diaphragm.?

We consider the substitution in the book of English for Latin terms, wherever practicable, an excellent feature. Apart from the advantage gained by the student, by the simplification of names, the sound and significance of which, hitherto, have so much monopolized the mind of beginners; we hail it as another step toward the explosion of a bubble—we have not the predilection of King James for the *communis lingua.*

For ourselves, we have adopted this book for review and dissections, and we find it a much less grave companion than our somewhat dingy, but indispensable Cruveilhier.

